# Interplay‐robust optimization for treating irregularly breathing lung patients with pencil beam scanning

**DOI:** 10.1002/mp.17821

**Published:** 2025-04-11

**Authors:** Ivar Bengtsson, Anders Forsgren, Albin Fredriksson, Ye Zhang

**Affiliations:** ^1^ Department of Mathematics KTH Royal Institute of Technology Stockholm Sweden; ^2^ RaySearch Laboratories AB Stockholm Sweden; ^3^ Center for Proton Therapy Paul Scherrer Institut Villigen Switzerland

**Keywords:** interplay‐driven optimization, motion mitigation, robustness

## Abstract

Background

The steep dose gradients obtained with pencil beam scanning allow for precise targeting of the tumor but come at the cost of high sensitivity to uncertainties. Robust optimization is commonly applied to mitigate uncertainties in density and patient setup, while its application to motion management, called 4D‐robust optimization (4DRO), is typically accompanied by other techniques, including gating, breath‐hold, and re‐scanning. In particular, current commercial implementations of 4DRO do not model the interplay effect between the delivery time structure and the patient's motion.

**Purpose**: Interplay‐robust optimization (IPRO) has previously been proposed to explicitly model the interplay‐affected dose during treatment planning. It has been demonstrated that IPRO can mitigate the interplay effect given the uncertainty in the patient's breathing frequency. In this study, we investigate and evaluate IPRO in the context where the motion uncertainty is extended to also include variations in breathing amplitude.

**Methods**: The compared optimization methods are applied and evaluated on a set of lung patients. We model the patients' motion using synthetic 4D computed tomography (s4DCT), each created by deforming a reference CT based on a motion pattern obtained with 4D magnetic resonance imaging. Each (s4DCT) contains multiple breathing cycles, partitioned into two sets for scenario generation: one for optimization and one for evaluation. Distinct patient motion scenarios are then created by randomly concatenating breathing cycles varying in period and amplitude. In addition, a method considering a single breathing cycle for generating optimization scenarios (IPRO‐1C) is developed to investigate to which extent robustness can be achieved with limited information. Both IPRO and IPRO‐1C were investigated with 9, 25, and 49 scenarios.

**Results**: For all patient cases, IPRO and IPRO‐1C increased the target coverage in terms of the near‐worst‐case (5th percentile) CTV D98, compared to 4DRO. After normalization of plan doses to equal target coverage, IPRO with 49 scenarios resulted in the greatest decreases in OAR dose, with near‐worst‐case (95th percentile) improvements averaging 4.2 %. IPRO‐1C with 9 scenarios, with comparable computational demands as 4DRO, decreased OAR dose by 1.7 %.

**Conclusions**: The use of IPRO could lead to more efficient mitigation of the interplay effect, even when based on the information from a single breathing cycle. This can potentially decrease the need for real‐time motion management techniques that prolong treatment times and decrease patient comfort.

## 1 INTRODUCTION

The ability of *pencil beam scanning* (PBS) to accurately shape dose distributions with the use of intensity modulation makes it the preferred delivery technique for proton radiation therapy treatments.^1^ However, this accuracy comes at the cost of high sensitivity to uncertainty in parameters affecting the dose delivery, such as tissue density, patient positioning, and patient motion. Robust optimization, which works by simultaneously considering and optimizing with respect to multiple error scenarios, has been clinically implemented to mitigate density‐ and positioning uncertainties.^2^, ^3^, ^4^ However, its application to motion management is typically complemented with active and/or passive motion management techniques during the treatment delivery.^5^, ^6^, ^7^ Active techniques, such as beam gating (with or without breath‐hold), are based on observing and then mitigating (and possibly controlling) the patient's motion. On the other hand, passive re‐scanning techniques mitigate motion by dividing the delivery of the plan into multiple sub‐deliveries, averaging out the dose degradation effects. All of the aforementioned motion management techniques suffer from drawbacks, either through prolonged treatment times or decreased patient comfort.

Current clinical implementations of robust optimization for motion mitigation, often called *4D‐robust optimization* (4DRO), extend the scenario definition from including a density scaling and a positioning shift to also including an anatomical state, typically represented by a4D computed tomography (4DCT) motion phase. The dose in a scenario is then computed under the assumption that all beam spots are delivered to a single phase. Consequently, a limitation of 4DRO is that it does not consider the interplay effect, which is the interference between the patient's motion and the time structure of the delivery.^8^, ^9^, ^10^, ^11^ Therefore, optimization models that consider the interplay effect are of research interest.

Previously, Bernatowicz et al. have proposed an optimization method that used *4D dose computations* (4DDCs) in the optimization, explicitly accounting for the interplay effect.^12^ Hereafter, we refer to this use of 4DDC in optimizing PBS plans as *interplay‐driven optimization* (IPO). Similarly, Graeff et al. have proposed a system that performs IPO and then uses the treatment control system to ensure the precise delivery of each pencil beam spot to the appropriate motion phase.^13^ The main challenge of IPO is analogous to the general uncertainty problem in PBS: failure to accurately represent the actual patient motion on the day of treatment leads to discrepancies between the intended and the realized treatment outcomes.

As a remedy to the problem of patient motion uncertainty, Engwall et al. have proposed *interplay‐robust optimization* (IPRO).^14^ This approach includes multiple patient motion scenarios in the optimization to represent the motion variability during the delivery. The study showed that IPRO can maintain robust target coverage to a greater extent than 4DRO. A limitation of their study was the reliance on a single pre‐treatment 4DCT used to model breathing motion in the numerical experiments. Consequently, the generated evaluation motion scenarios were based on only ten motion states per patient, varying in the period of each breathing cycle. Therefore, although IPRO was shown to mitigate uncertainty in patient breathing frequency, its ability to mitigate uncertainty related to variation in breathing amplitude remains to be demonstrated.

In this work, we extend the investigation of IPRO to the case of irregular breathing, in which the patient sequentially passes through distinct breathing cycles, varying in frequency and peak amplitude. Our breathing model is based on motion states that were previously synthesized in the work by Duetschler et al.,^15^ which used a reference CT and deformation vector fields acquired with 4D magnetic resonance imaging (4DMRI) to generate synthetic 4DCTs (s4DCT), spanning multiple breathing cycles varying in period and amplitude. The optimization method used considers doses computed by 4DDC using phase sorting^10^, ^14^, ^16^, ^17^ extended to irregular motion. For evaluation, the delivered doses are computed analogously on breathing data unseen at the optimization stage. This way, we aim to answer if IPRO can provide dosimetric advantages over 4DRO in the case of irregular breathing, where the patient's motion during delivery is only partially known during the treatment plan optimization.

## 2 METHOD

This work uses a time‐varying volumetric image to represent the patient's motion. In particular, this image is represented by a s4DCT. A snapshot of the s4DCT at a particular time is called a *motion state*. Our 4DDCs follow a phase‐sorting‐based approach, in which partial doses are computed on each motion state depending on the exact delivery times of each pencil beam spot and accumulated on a reference state. We then perform IP(R)O considering doses computed with 4DDC.

### 2.1 4DDC

Consider an index set P that enumerates all plausible motion states of the patient. A plausible motion pattern of the patient during the delivery of a treatment fraction is called a *motion scenario* and is described by a function s(t) that maps time points to motion states, $s:R_{+}\to P$.

Then consider the set $I^{p}\left(x;s\right)$ as the indices of the spots delivered on state p given for the spot weights x in scenario s. Given the dose‐influence matrix $D^{p}$ that encodes in its columns the dose of each pencil beam as if delivered during state p, the partial dose $d^{p}\left(x;s\right)$ can be computed considering only the relevant columns of $D^{p}$:

(1) $$d^{p}\left(x;s\right)=D^{p}{\tilde{x}}^{p}\left(x;s\right),$$

where the auxiliary variable ${\tilde{x}}^{p}\left(x;s\right)$ is defined from

(2) $${\tilde{x}}_{i}^{p}\left(x;s\right)=\left\{\begin{array}{c} x_{i}\;\text{if}\;i\in I^{p}\left(x;s\right),\\ 0\;\text{otherwise.} \end{array}\right.$$

The fraction dose is then the accumulation of the deformed partial doses:

(3) $$d\left(x;s\right)=\sum_{p\in P}R^{p}d^{p}\left(x;s\right),$$

where each $R^{p}$ is a matrix—resulting from deformable registration—that encodes the dose deformation from a state p to the reference state.

### 2.2 IPRO

The main principle of IPRO is to minimize an objective function that takes as input the doses computed with 4DDC considering scenarios in a set S:

(4) $$\underset{x\;\in \;X}{\text{minimize}}\;\;\underset{s\in S}{\max\;}f\left(d\left(x;s\right)\right),$$

where X is the set of feasible spot weights. Here, we recognize that in the typical case of the delivery time structure for PBS, the 4D‐computed dose d(x;s) is a nonlinear and non‐convex function in x, stemming from the fact that weights of earlier spots will influence the start time of later spots. However, as previous studies have successfully mitigated this non‐linearity heuristically by keeping the set $I^{p}\left(x;s\right)$ fixed in x and updating it regularly during the numerical optimization process, we will employ the same strategy in this work.^12^, ^14^ Importantly, IPO can be seen as a special case of Problem (4) with S as a set containing a single scenario.

### 2.3 Data

Our 4DDCs depend on the time structure of the PBS delivery, the considered motion states, and the order in which they appear in each motion scenario.

#### 2.3.1 Delivery time structure

The delivery time structure was given by a model that describes the dose rate, the lateral scanning time, and the energy switching time – all based on fits of the experimental data from an IBA ProteusPlus system (IBA, Louvain‐La‐Neuve, Belgium) in Pfeiler et al.^18^ The dose rate, measured in milliseconds per monitor unit, was replicated exactly as the model fit in their Figure 6: $\frac{2588.986}{{\left(MU+1.256\right)}^{16.721}}+4.995$ (ms/MU). The lateral scanning times were approximated by a linear fit of the data points in their Figure 7. For consecutive spots with lateral coordinate absolute differences (Δy,Δz), the scan time was computed as max{0.3125Δy+2.2187,3.1250Δz+1.9375}. Finally, the energy switching time was a constant 1230 ms.

#### 2.3.2 s4DCT generation

The s4DCTs employed in this study were previously generated in the work by Duetschler et al.^15^ The method works by deforming a reference CT with the deformable vector fields produced by the deformable registration of motion acquired with 4DMRI at 2.25 Hz. With a 4DMRI of sufficient duration, one can generate enough motion states to cover the duration of a PBS treatment. For a more detailed description of the s4DCT used, the reader is referred to the original paper.^15^

#### 2.3.3 Patient cases and motion patterns

Three distinct geometries were considered using three reference CTs of non‐small cell lung cancer patients (CT 1, 2, and 3). In CT 1, the CTV had a volume of 376.59 ${\text{cm}}^{3}$ and was located in the superior lobe, lying on the horizontal fissure. It had a large, mainly posterior, attachment to the thoracic wall. For CT 2, the CTV was in the mediastinum and had a volume of 106.19 ${\text{cm}}^{3}$. Finally, CT 3 had a peripheral tumor, located in the posterior direction in the left inferior lobe. The CTV volume was 51.64 ${\text{cm}}^{3}$.

**FIGURE 1:**
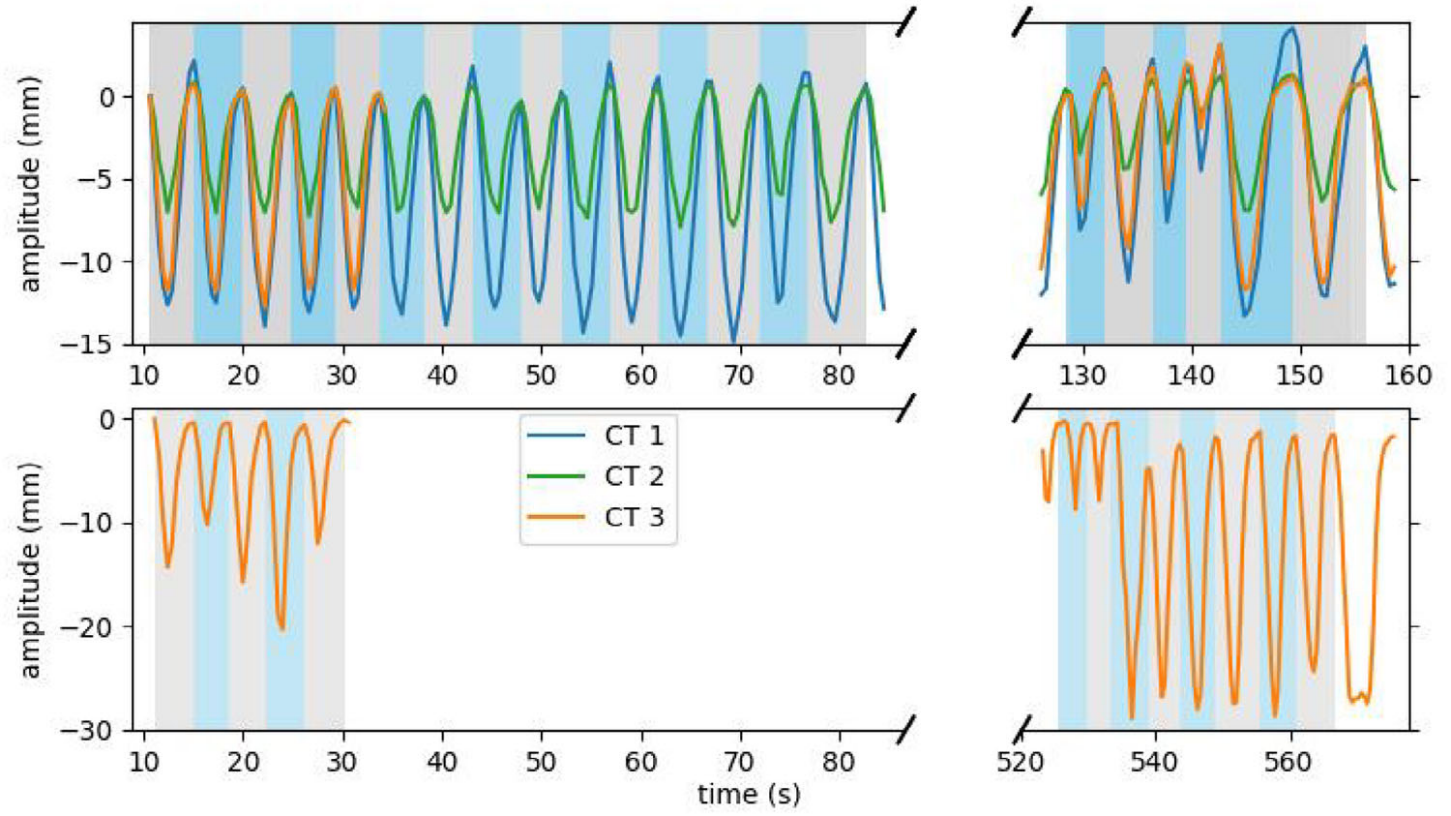
The motion patterns (amplitude (mm) over time (s)) in the four cases. The motion pattern from 4DMRI 1 (top) is considered for all three reference CTs, while that from 4DMRI 2 (bottom) is considered only for CT 3. The background is shaded to distinguish individual breathing cycles and to indicate the partitioning of the set of breathing cycles into one subset for optimization (odd indices/ gray) and one for evaluation (even indices/ blue).

All geometries were used to make s4DCTs using a single breathing pattern from a healthy volunteer (4DMRI 1), yielding three unique cases: case 1, case 2, and case 3a. A second breathing pattern (4DMRI 2) was considered for CT 3 to generate a fourth case, referred to as case 3b. Although the same 4DMRI was used for cases 1, 2, and 3a, the motion magnitudes of the tumors vary, depending on their position within the lung. The four motion patterns are shown in Figure 1. For all cases, the motion was predominantly in the inf‐sup dimension. Therefore, we estimate the motion magnitude as the inf‐sup‐coordinate of the deformation vector in the center of the CTV.

### 2.4 Scenario modeling

After identifying the peaks of the motion signal, we defined distinct breathing cycles by grouping the motion states between neighboring peaks (corresponding to the end‐exhale phase). This process produced a set of breathing cycles per patient, each comprising 7–15 motion states. The purpose of this grouping was to allow for the generation of a large and varied set of plausible breathing scenarios per patient. Since the motion patterns were acquired at a constant rate of 2.25 Hz, the scenario function s was implicitly defined by an ordered sequence of motion states of sufficient duration to cover the treatment delivery.

We then partitioned the set of breathing cycles into two subsets: one for plan optimization and the other for evaluation of plan delivery. Both motion patterns consisted of sub‐patterns acquired during distinct time intervals. Therefore, the partition was made to avoid bias toward a specific breathing trend by selecting every other breathing cycle for the optimization set (odd indices) and the rest for the evaluation set (even indices). This selection is visualized by the varying background shades in Figure 1, and two resulting sets of breathing cycles are further described in Table 1.

**TABLE 1:** Statistics for each case.

			Optimization set				Evaluation set			
Case	CTV volume (${\text{cm}}^{3}$)	CTV location	Count	Period (s)	Max. disp. (cm)	Min. disp. (cm)	Count	Period (s)	Max. disp. (cm)	Min. disp. (cm)
1	376.59	Superior lobe, pleural attachment	11	4.85±0.94	0.13±0.11	−1.24±0.27	10	4.62±0.89	0.13±0.11	−1.22±0.23
2	106.19	Mediastinum	11	4.81±0.89	0.06±0.04	−0.63±0.18	10	4.67±0.89	0.06±0.04	−0.63±0.16
3a	51.64	Left interior lobe, peripheral nodule	6	4.44±0.68	0.10±0.06	−0.98±0.37	5	4.44±1.32	0.13±0.11	−0.96±0.27
3b	−	−	7	4.57±1.11	−0.11±0.10	−1.84±0.72	6	4.74±0.80	−0.08±0.08	−2.08±0.85

*Note*: The leftmost columns inform about the size and placement of the CTV and the rightmost about the breathing cycles selected for optimization (odd indices) and evaluation (even indices), respectively. Each quantity describing breathing is expressed using the mean and standard deviation (μ±σ) over the selected set of breathing cycles.

**FIGURE 2:**
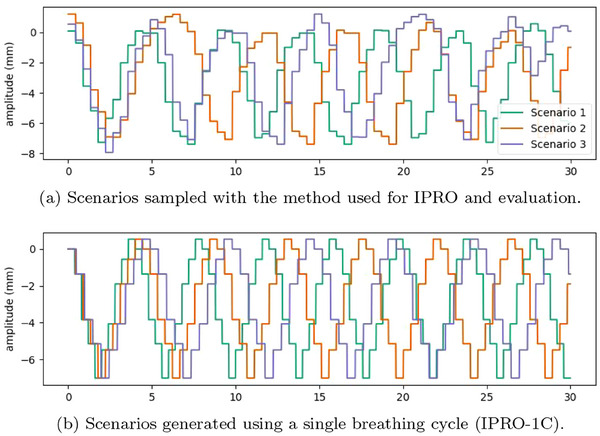
Amplitudes corresponding to the first 30s of three scenarios for patient 2.

We generated each evaluation motion scenario by randomly concatenating breathing cycles from the evaluation set. For each beam, the motion state sequence was constructed by drawing (with uniform probability and with replacement) and concatenating breathing cycles until the total breathing time corresponding to the sequence was sufficient to cover the duration of the beam delivery. Due to the variation in period between the breathing cycles, this method implicitly generates phase variation within the set of generated breathing patterns and allows for studying the interplay effect under various motion scenarios. It implicitly relies on the assumption that there is no uncertainty about the breathing cycle phase at the start of the delivery of each beam, as each breathing cycle begins in the end‐exhale phase. Figure 2a shows three scenarios generated using the described method.

### 2.5 Treatment evaluation

We evaluated the robustness of each investigated treatment plan by simulating the plan delivery–without gating or re‐scanning–in multiple motion scenarios, generated as described in Section 2.4. The time structure of the delivery was as described in Section 2.3.1. Combining a motion scenario, a treatment plan, and its corresponding time structure enabled 4DDC with the methodology described in Section 2.1. The number of evaluation motion scenarios per plan was 500, as recommended by Pastor‐Serrano et al. for statistically accurate analysis of dose uncertainties in the presence of interplay.^19^ Consequently, we analyzed 500 dose distributions per investigated plan by their variation of various dosimetric qualities and the objective function value.

### 2.6 Treatment planning

Treatment planning was performed using a RayStation (RaySearch Laboratories AB, Stockholm, Sweden) machine model of an IBA system with the Dedicated PBS nozzle. All compared plans originated from the same initial *template plan*. This plan included three beams with angles chosen based on the patient's geometry. It was optimized with 40 iterations of 4DRO,^3^ with the scenarios being the motion states from a full breathing cycle containing the reference CT. Setup and range uncertainties were not considered. The template plan acted as a basis for all the compared plans in three ways: First, it distributed sufficiently many spots around the CTV to account for the full range of motion and ensured that all compared plans used the same set of spots. Second, it allowed the selection of objective functions and weights that complied with the treatment's dosimetric goals. Third, it provided an improved and consistent initial point for the numerical algorithm employed in the optimization of the compared plans.

The objective functions used in the optimization of all plans, including the template plan, were designed per patient to comply with the dosimetric criteria from RTOG 1308,^20^ but limited to the available OAR segmentations, and scaled to a single fraction with 200 cGy as the prescribed dose. To obtain target coverage, we increased the objective weight of the CTV from an initial guess until the CTV D99 reached the prescribed dose in all motion states or until the weight increases did not result in any further improvements (which occurred for one CT when the CTV D99 was 99.5% of the prescribed dose). In the case where OAR objectives were used, they were designed to penalize dose above the dose levels specified in the criteria from RTOG 1308. Due to a mismatch between the segmented OARs and those listed in RTOG 1308, an objective was used for the medulla instead of the spinal cord for CT 1. A complete list of objectives used for each CT geometry can be found in Appendix A.

### 2.7 Optimization methods

All optimization methods employed 40 iterations of sequential quadratic programming (SQP), with the initial point as the spot weights from the template plan and without any further spot weight filtering.

- **4D Robust (4DRO)**: This plan was generated by 40 additional iterations of the SQP solver in RayStation and acted as a reference for the results of the interplay‐driven plans.

Except for 4DRO, the investigated plans were all optimized using IPRO and differ only in the scenario set S, under different assumptions on the availability of representative breathing data. They were optimized using 40 iterations of the SQP solver SNOPT (7.7 Stanford Business Software, Stanford, California), interfaced with Matlab (2024a, Mathworks Inc., Natick, Massachusetts, USA). To smoothen the objective in Problem (4), the max‐operator was approximated with the weighted‐power‐mean operator with the power parameter set to 8.

- **Non‐robust IPO**: To study the performance of plans optimized considering a single breathing cycle, we implemented IPO with the breathing scenario taken as the repetition of the first breathing cycle available in the data.
- **IPRO with repeated breathing (IPRO‐1C)**: Because it may be infeasible to acquire motion states from multiple breathing cycles for treatment planning, this first IPRO method used a scenario set consisting only of scenarios which were repetitions of the first breathing cycle, shifted in start phase and scaled in breathing period. Three sets of scenarios were investigated: one using breathing period scaling factors in {0.9,1,and1.1} and start phase shifts in {−1,0,and+1} (9 scenarios), the second using breathing period scaling factors in {0.8,0.9,1,1.1,and1.2} and start phase shifts in {−2,−1,0,+1,and+2} (25 scenarios), and the third with breathing period scaling factors in {0.7,0.8,0.9,1,1.1,1.2,and1.3} and start phase shifts in {−3,−2,−1,0,+1,+2,and+3} (49 scenarios).
- **IPRO with simulated breathing (IPRO)**: To study the extent to which further robustness gains can be made by including additional breathing cycles in the optimization, we considered a scenario set generated by the same method as the set of evaluation scenarios but by randomly selecting breathing cycles for concatenation from the optimization set. This method was investigated for 9, 25, and 49 scenarios.

A challenge of IP(R)O(‐1C) is that the delivery time structure changes with the variable spot weights. As in previous literature,^12^, ^14^ we address this heuristically by updating the delivery time structure according to the current spot weights at regular intervals (every 10 iterations) during the optimization process.

## 3 RESULTS

Here, we present the performance of each planning method in each of the investigated patient cases. Performance is measured by considering the variation in dosimetric criteria over the evaluated motion scenarios. For the different cases, we show in Figure 3a–o boxplots of the CTV D98, the CTV homogeneity index (HI = $\frac{\text{D2 - D98}}{\text{D50}}$), and dose to a relevant OAR. Each figure also shows the variation of the considered metric between the phase doses used in the optimization of 4DRO (each deformed to the planning image). These *static* doses are unrealistically good for two reasons: they are computed without considering the interplay effect, and they are evaluated on the same images as they were optimized (unlike the evaluated methods). Still, they indicate the ideal performance and are included for reference.

FIGURE 3Distributions of CTV and medulla dose statistics for each of the investigated optimization methods for cases 1 and 2. The boxes indicate the inter‐quartile range, while the whiskers indicate the 5th and 95th percentiles. For each box, the distribution mean and median are indicated by the plus and the solid line, respectively. Additionally, the shaded area specifies the spread of values among the optimized phase doses achieved by 4DRO (deformed to the planning image) and acts as an indication of the desired dosimetric trade‐offs. Distributions of CTV and OAR dose statistics for each of the investigated optimization methods for cases 3a, 3b, and 3b* (in which the alternative breathing cycle was used for IPRO‐1C). 4DRO, 4D‐robust optimization; IPRO, interplay‐robust optimization.

**Figure:**
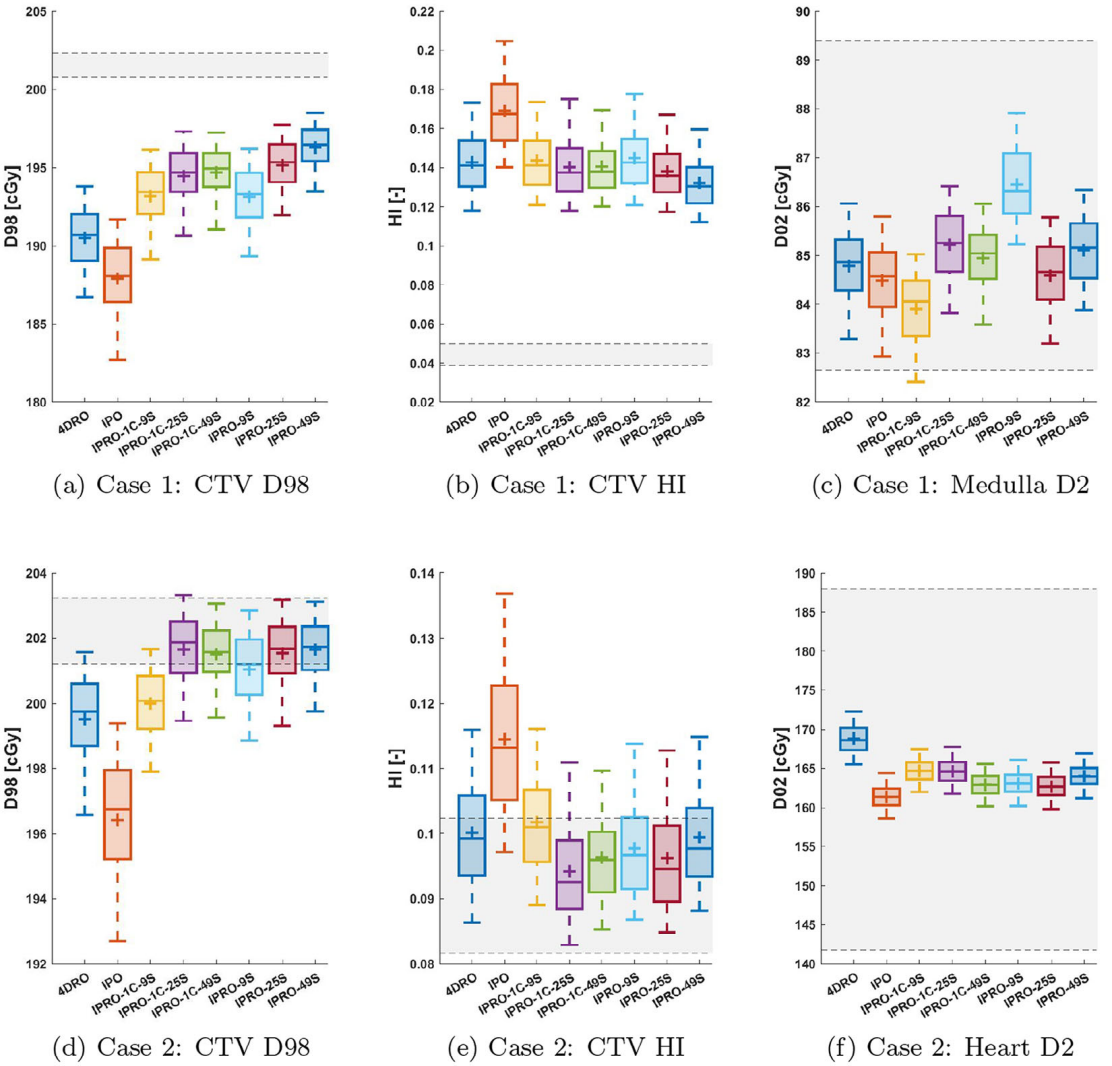

**Figure:**
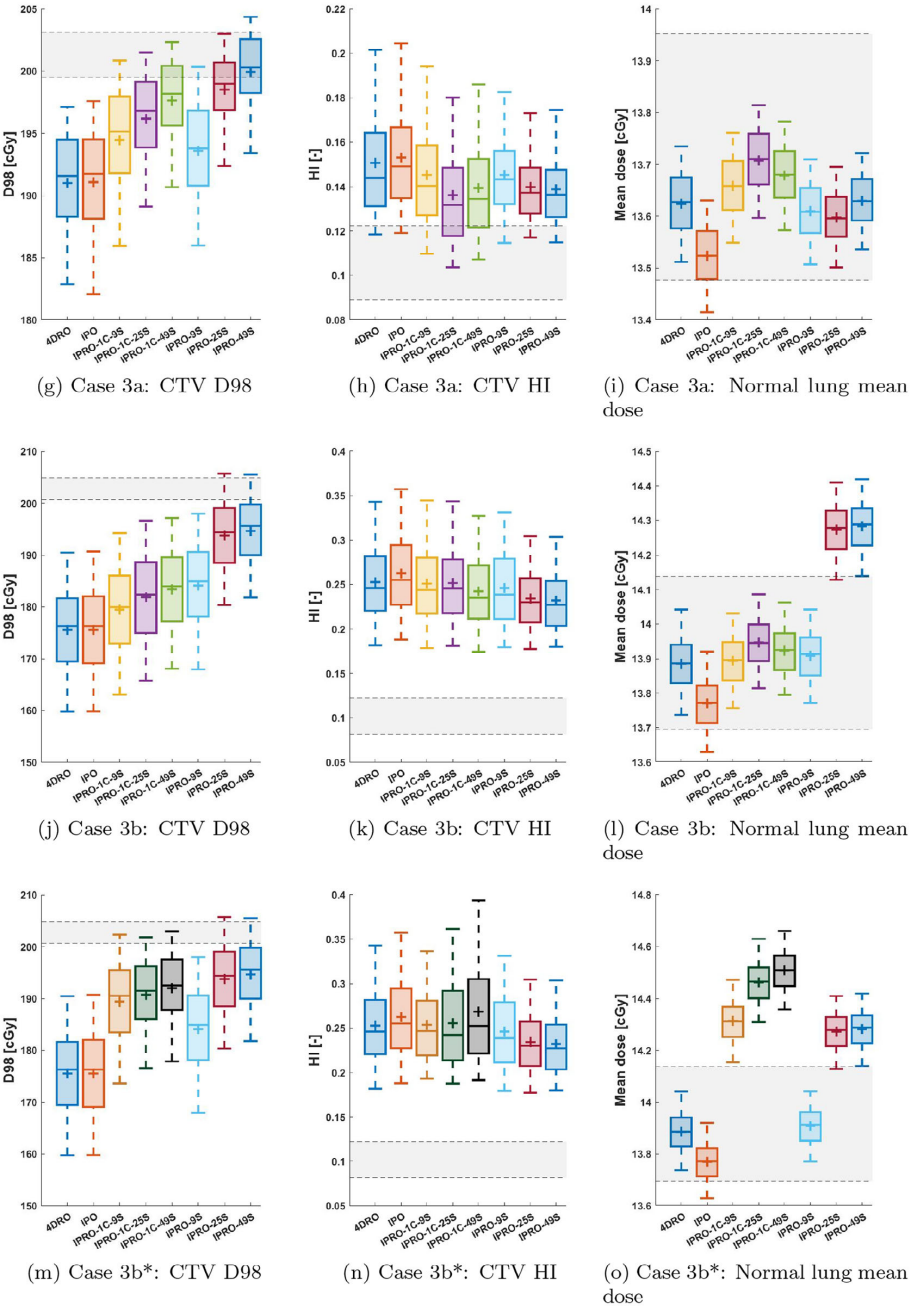

Because of the different implicit trade‐offs made in the optimization between conflicting objectives, it is difficult to determine the most favorable method from the unprocessed results. To address this issue, we also present doses that are normalized to have equal near‐worst‐case target coverage. More precisely, the evaluation doses were scaled by a factor to make the 5th percentile D98 equal to that of the evaluation doses for the 4DRO, acting as the benchmark. For simplicity, it was assumed that the scaling could be performed with negligible effect on the delivery time structure. The 95th percentile OAR doses, as well as the CTV D2, after normalization, are then shown in Table 2.

**TABLE 2:** The near‐worst‐case (95th percentile) patient‐specific dosimetric results after normalizing the doses for each method to match the 5th percentile CTV D98 for 4DRO.

Case	ROI	Metric	4DRO (cGy)	IPO	IPRO‐1C‐9S	IPRO‐1C‐25S	IPRO‐1C‐49S	IPRO‐9S	IPRO‐25S	IPRO‐49S
				Change (%) relative to 4DRO						
1	CTV	D2	223.4	+3.8	+0.1	+0.1	−0.4	+0.4	−0.8	− **{1.7}**
	Heart	D2	61.3	+0.9	−1.9	−2.5	−2.4	−1.7	−3.2	− **4.2**
		Mean	3.8	+0.9	−1.6	−2.0	−1.9	−1.6	−2.8	− **3.7**
	Medulla	D2	86.1	+1.9	−2.5	−1.7	−2.3	+0.7	−3.0	− **3.2**
		Mean	7.7	+0.7	−2.9	−2.8	−3.1	−1.5	−3.9	− **4.2**
	Esophagus	D2	202.5	+2.0	−0.7	−2.5	−2.3	−1.9	−2.6	− **3.0**
		Mean	32.7	+2.7	+0.4	−1.4	−1.0	−0.9	−1.4	− **2.0**
	Normal lung	D2	206.8	+1.9	−1.1	−1.5	−1.7	−0.9	−2.0	− **2.7**
		Mean	30.5	+0.4	−2.5	−2.9	−3.2	−2.4	−3.5	− **4.3**
2	CTV	D2	222.7	+1.9	−0.2	− **0.9**	− **0.9**	−0.8	−0.7	−0.6
	Heart	D2	172.2	−2.6	−3.4	−4.0	− **5.3**	−4.7	−5.1	−4.6
		Mean	11.4	−0.1	−0.9	−2.2	− **2.9**	−2.1	−2.5	−1.4
	Normal lung	D2	164.2	−0.1	−2.6	− **3.3**	−3.2	−2.9	−2.9	−3.2
		Mean	21.6	−0.6	−2.8	−3.4	− **3.6**	−3.2	−3.2	−3.4
3a	CTV	D2	230.2	+0.5	−0.2	−2.1	−1.6	−1.6	− **3.1**	−2.6
	Normal lung	D2	188.7	+0.8	−0.5	−1.9	−2.8	−1.2	− **4.5**	− **4.5**
		Mean	13.7	−0.3	−1.5	−2.7	−3.8	−1.8	−5.2	− **5.5**
3b	CTV	D2	238	+1.6	+0.7	−0.2	−1.8	−1.0	−3.4	− **3.6**
	Normal lung	D2	185.2	−0.4	−1.1	−2.1	−3.6	−3.5	−7.5	− **8.0**
		Mean	14	−0.9	−2.1	−3.3	−4.8	−4.9	−9.1	− **9.7**
3b*	CTV	D2	−	−	+0.5	+1.1	+2.5	−	−	−
	Normal lung	D2	−	−	−4.5	−4.7	−4.7	−	−	−
		Mean	−	−	−5.2	−5.7	−6.2	−	−	−

*Note*: Red and green cells indicate an increase or decrease by 1% or more with respect to 4DRO, respectively. The prescribed dose was 200 cGy. The **bold** values indicate the best‐performing method with respect to the relevant metric. The asterisk indicates the version of case 3b with a different breathing cycle used for IPRO‐1C.

Abbreviations: 4DRO, 4D‐robust optimization; IPRO, interplay‐robust optimization; IPO, interplay‐driven optimization.

Another way to compare the methods without having to consider trade‐offs between conflicting criteria is to compare the impact on the objective function used in optimization. Therefore, we show the variations in objective value over the evaluation scenarios, without normalization, in Appendix B.

### 3.1 Case 1 − Large CTV

CT 1 had the largest CTV, resulting in plans with 105 energy layers and 12716 spots. The dosimetric performance is shown in Figure 3a–c, where Figure 3c shows the D2 to the medulla, the OAR contributing the most to the objective function (Table A1).

**TABLE A1:** The patient‐specific objectives used to optimize plans for all methods.

Geometry	ROI	Function type	Dose level (cGy)	Weight
CT 1	CTV	Min. dose	201	400
	CTV + 1cm	Max. dose	210	50
	Heart	Max. dose	86	1
	Heart	Max dose	129	1
	Medulla	Max. dose	143	100
	Esophagus	Max. dose	211	1
CT 2	CTV	Min. dose	201	1200
	CTV + 1 cm	Max. dose	210	10
	Heart	Max. dose	86	1
	Heart	Max. dose	129	1
CT 3	CTV	Min. dose	201	80
	CTV + 1cm	Max. dose	210	1

Compared to the plan made with 4DRO, acting as the benchmark, the plans optimized with IPRO(‐1C) consistently increased the CTV D98 in both the mean and 5th percentile. This was achieved without a clear increase to the CTV HI or the medulla mean dose. In contrast, IPO failed to match the CTV D98 or the CTV HI of 4DRO. Considering the effect of IPRO(‐1C) after normalization, the CTV D2 was not drastically affected; only IPRO‐49S changed by more than ±1%. The effect on OAR dose was more apparent, with typical decreases by more than 1% for most IPRO(‐1C) methods, and IPRO‐49S consistently performing the best with decreases in mean dose by 2.0%–4.3% and in D2 by 2.7%–4.2%.

### 3.2 Case 2 − Smaller motion amplitude

Case 2 required 80 energy layers and 6834 spots to cover the CTV. The motion amplitude was approximately half that of the other cases, suggesting less severe consequences of the interplay effect. This was confirmed by the CTV D98, which was considerably higher than for the other patient cases, as shown in Figure 3d–f. For example, the mean and median of IPRO(‐1C) with 25 or more scenarios were inside the interval based on the 4DRO static doses.

Considering the normalized doses (Table 2), all the interplay‐based methods decreased the heart D2 by at least 2.6%. For the robust methods, the decrease was 3.4%–5.3%, with the greatest decrease from IPRO‐1C‐49S. For normal lung, IPRO(‐1C) decreased D2 (mean) by 2.6%–3.3% (2.8%–3.6%), while the corresponding decrease for IPRO was 2.9%–3.2% (3.2%–3.4%).

### 3.3 Case 3a − Small CTV

CT 3 had the smallest CTV, requiring only 54 energy layers and 3439 spots distributed across the three beams. The size of the CTV made treatment planning more difficult, resulting in greater dose heterogeneity for all plans. As optimization objectives were applied only to the CTV, the mean dose to the normal lung (both lungs minus CTV) is shown alongside the CTV statistics in Figure 3g–i to indicate the dosimetric effect outside the CTV.

IPRO(‐1C) consistently improved the mean and near‐worst‐case for both CTV D98 and CTV HI compared to 4DRO. After normalizing the doses, IPRO with 25 and 49 scenarios performed similarly (Table 2), improving CTV D2 by 2.6%–3.1% and normal lung D2 and mean dose by 4.5% and 5.2%–5.5%, respectively.

### 3.4 Case 3b − Second motion pattern

The results for case 3b, with the second motion pattern, are shown in Figure 3j–l. As seen in Figure 1, the second motion pattern differs from the first in that the first breathing cycle has much less amplitude variation than the maximum variation over all cycles within the motion pattern. Consequently, the difference between IPRO‐49S, which performed the best in CTV D98 and CTV HI, and IPRO‐1C‐49S was larger than for the other cases. Therefore, we repeated the experiment, calling it 3b*, with another breathing cycle to generate the scenario set for IPRO‐1C. The consequence, shown in Figure 3m–o, was a clear increase in CTV D98 and normal lung mean dose.

The normalization of target doses (Table 2) shows that IPRO‐49S performed the best, even after the adjustments to the planning breathing cycle used for IPRO‐1C, reducing the CTV D2, the normal lung D2, and the normal lung mean dose by 3.6%, 8.0%, and 9.7%, respectively. However, the effect of the change of breathing cycle was apparent regardless of the number of scenarios. In particular, IPRO‐1C‐9S improved in every metric and achieved results comparable to IPRO‐9S.

## 4 DISCUSSION

We implemented IPRO for patient cases with synthetically generated irregular breathing motion. The results show that IPRO consistently improves both mean and near‐worst‐case CTV D98, with little effect on CTV dose homogeneity, for PBS deliveries in the investigated cases. In most cases, these results are achieved without compromising OAR‐sparing. In addition, the OAR doses−whose functions were typically of low weight in the optimization objective−typically varied little compared to their variation within the static doses used in 4DRO. In some cases where IPRO(‐1C) substantially improved the target coverage, the integral dose (measured by the mean dose in the normal lung and not included in the optimization) increased to be outside of the interval spanned by the static doses (Figure 3l). However, there were also situations where the OAR dose could be simultaneously decreased while improving the target dose. This was the case for the heart D2, shown in Figure 3f, where all IPRO(‐1C) methods achieved lower values, although they also all had improved the CTV D98 and most had improved the CTV HI (Figure 3a‐b). Our interpretation of this favorable behavior of IPRO(‐1C) is that the 4DDC used for optimization more accurately represents the dose that will be delivered, which is exploited by the optimization of intensity modulation to find more efficient trade‐offs between conflicting objectives, compared to 4DRO which optimizes considering statically computed dose distributions. In addition, when normalizing all methods to equal 4DRO in target coverage, the OAR‐sparing effects of IPRO(‐1C) were apparent (Table 2). This result is most apparent for IPRO, based on optimization scenarios sampled using the same technique used to sample evaluation scenarios. The result also holds−although with smaller improvements−for IPRO‐1C, based on using a single breathing cycle to generate the optimization scenarios deterministically.

Between the scenario generation methods for IPRO(‐1C), there was typically a positive impact from increasing the number of scenarios, with few exceptions. Likewise, using multiple breathing cycles to generate the optimization scenarios (IPRO) was advantageous compared to using a single cycle. This difference was most apparent for case 3b, for which the improvement in 5th percentile CTV D98 with IPRO‐1C was less than half of that from using IPRO, which showed that IPRO‐1C with a single breathing cycle unrepresentative of the evaluation motion scenarios may fail to generate robust plans. However, we then showed (with Case 3b*) that this discrepancy can be decreased by using a breathing cycle with sufficient amplitude range (Figure 3m–o).

A limitation of this work is the disregard of ribcage motion in the synthetization of motion states. This follows from the method used to generate s4DCTs in Duetschler et al.^15^ Furthermore, breathing patterns from healthy volunteers may not be the most accurate representation of the breathing patterns for non‐small cell lung cancer patients. Finally, the motion pattern of a lung tumor may vary depending on its location within the lung, as presented by Seppenwoolde et al.^21^ Consequently, although our study has generalized the results from Engwall et al.^14^ to irregular motion, further work that investigates IPRO with an even more realistic representation of patient breathing motion is of interest. We believe that the improvement presented by IPRO over 4DRO would hold regardless of the exact patient breathing model as long as it is consistent between optimization and evaluation.

Instead, the case that may compromise the robustness aimed at by IPRO is when the motion states that may occur during treatment delivery are not represented in the scenarios used during optimization. This was the case with the initial results for IPRO‐1C for case 3b and it is possibly also the reason for the performance difference between IPRO with 9, 25, and 49 scenarios. In this study, we selected the optimization and evaluation sets to be unique but statistically similar (Table 1). Larger discrepancies between the data used for optimization and evaluation, respectively, are expected to decrease the robustness gain of IPRO. Non‐robust IPO was included in the numerical experiments primarily to highlight this effect; the accuracy of PBS allows highly conformal plans that are very sensitive to delivery uncertainty. To further improve IPRO‐based treatment planning, work is needed to establish the most appropriate representation of the patient's motion, including uncertainties.

There are also practical considerations needed to enable a clinical implementation. First, in current clinical practice, the exact time structure of the delivery is typically not available during treatment planning. To implement IPRO, this information needs to be made available to the treatment planning system. In our study, we have estimated the time structure using a simple model based on experimental data. In the previous study by Engwall et al., the delivery time structure was estimated by a simulation using the IBA tool ScanAlgo.^14^ Preferably, machine‐specific information would be provided by the manufacturers through the treatment control system and communicated to the treatment planning system. Regardless of the exact implementation, the robustness of the dose to uncertainties about the time structure should be considered. Second, the computational demands of representing a large set of breathing scenarios and the associated dose‐influence matrices posed a challenge in this study. The development of faster and more memory‐efficient methods for dose computation, motion modeling, and optimization may be needed to accurately represent, evaluate, and mitigate patient motion on current hardware. To clarify, IPRO‐1C‐9S is not a more computationally intensive task than 4DRO; it uses the same number of motion states and considers fewer scenarios (nine rather than ten). Instead of contour sets on each of the included motion states, deformable registrations are needed to accumulate the dose on the reference state. However, the other IPRO methods all have computational disadvantages, either from using more scenarios or from the increased memory requirements associated with storing the dose‐influence matrices of more than 10 motion phases. IPRO‐1C can be implemented without the increased memory requirements, and the computational time increases approximately linearly since the computational effort is dominated by performing the matrix‐vector products related to calculating the scenario doses and the objective gradients. The increased memory requirements associated with IPRO, however, limited our analysis to a maximum of 49 scenarios before the computations on our desktop computer slowed down severely. This may challenge the implementation of IPRO on larger tumors.

This study was delimited to consider the planning and delivery of a single treatment fraction. The errors associated with breathing motion are inherently random rather than systematic, and an important direction of future work is to investigate the methods in the presence of other processes that mitigate the interplay effect, such as re‐scanning and fractionation. Whether the indicated advantages of IPRO translate into a clinical benefit can only be established if the comparison is made considering such conventional clinical practices and other relevant uncertainty sources. Therefore, interesting future work includes comparing the methods in the presence of re‐scanning and fractionation. For example, an interesting question is if there is a number of re‐scans for which the motion‐robustness properties of IPRO and 4DRO are indistinguishable. Once such an investigation has been made, it may also be relevant to investigate alternative formulations of the IPRO problem, that explicitly take re‐scanning and fractionation into account in the dose calculation used for optimization. This way, their cancellation effects would be included in the optimization model and potentially allow for the reduction of OAR and integral dose. Alternatively, the higher interplay‐robustness of IPRO could likely be used to alleviate some of the need for either re‐scanning, breath hold, or gating, leading toward treatments of shorter duration and increased patient comfort, or increasing the potential of hypofractionated treatments.

## 5 CONCLUSION

In this study, we implemented different variants of IPRO and evaluated the resulting plans based on synthetically generated 4DCTs exhibiting irregular breathing. The findings indicate advantages of using IPRO over conventional 4DRO. Including more scenarios and breathing cycles in the optimization typically led to the largest dosimetric improvements, highlighting the importance of accurately representing the breathing variation. However, the explicit consideration of the interplay effect by generating breathing scenarios using a single breathing cycle (IPRO‐1C) already presents improvements over 4DRO.

## CONFLICT OF INTEREST STATEMENT

Ivar Bengtsson and Albin Fredriksson are employed by RaySearch Laboratories.

## Figures and Tables

**FIGURE B1:**
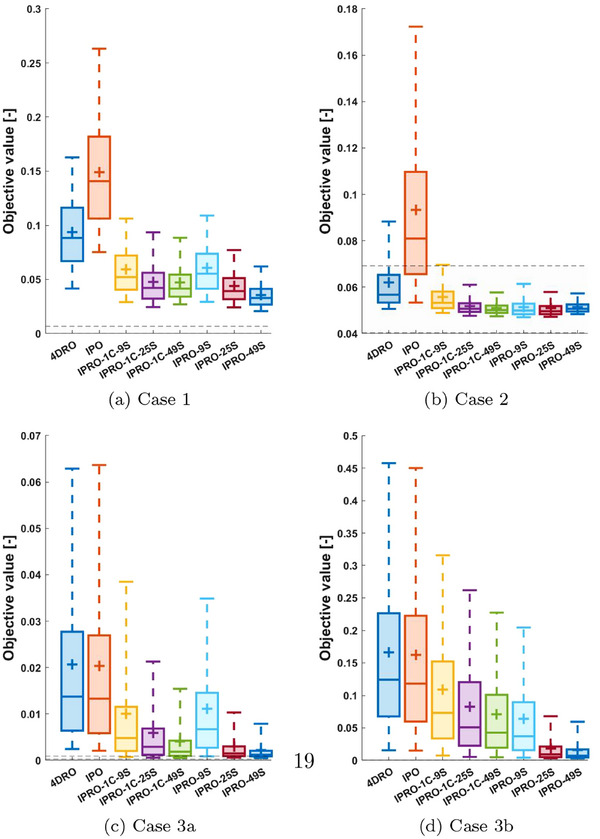
The distribution of objective function values over the evaluation motion scenario for each method and case.

## APPENDIX A OPTIMIZATION OBJECTIVE FUNCTIONS

The geometry‐specific objective functions are presented in Table A1.

## APPENDIX B OBJECTIVE VALUES

Figure B1 shows the distribution of objective function values for each method and case.
